# How the introduction of OSCEs has affected the time students spend studying: results of a nationwide study

**DOI:** 10.1186/s12909-019-1570-6

**Published:** 2019-05-15

**Authors:** Stefan Müller, Ines Koch, Utz Settmacher, Uta Dahmen

**Affiliations:** 1 Department of General, Visceral and Vascular Surgery, Universitätsklinikum Jena, Am Klinikum 1, 07747 Jena, Germany; 2 Department of Gynaecology and Reproductive Medicine, Universitätsklinikum Jena, Am Klinikum 1, 07747 Jena, Germany; 3 Department of General, Visceral and Vascular Surgery, Experimental Transplantation Surgery, Universitätsklinikum Jena, Drackendorfer Str. 1, 07747 Jena, Germany

**Keywords:** Assessment, OSCEs, Study behaviour, Medical students, Survey

## Abstract

**Background:**

Medical schools globally now use objective structured clinical examinations (OSCEs) for assessing a student’s clinical performance. In Germany, almost all of the 36 medical schools have incorporated at least one summative OSCE into their clinical curriculum. This nationwide study aimed to examine whether the introduction of OSCEs shifted studying time. The authors explored what resources were important for studying in preparation for OSCEs, how much time students spent studying, and how they performed; each compared to traditionally used multiple choice question (MCQ) tests.

**Methods:**

The authors constructed a questionnaire comprising two identical sections, one for each assessment method. Either section contained a list of 12 study resources requesting preferences on a 5-point scale, and two open-ended questions about average studying time and average grades achieved. During springtime of 2015, medical schools in Germany were asked to administer the web-based questionnaire to their students in years 3–6. Statistical analysis compared the responses on the open-ended questions between the OSCE and MCQs using a paired *t*-test.

**Results:**

The sample included 1131 students from 32 German medical schools. Physical examination courses were most important in preparation for OSCEs, followed by class notes/logs and the skills lab. Other activities in clinical settings (e.g. medical clerkships) and collaborative strategies ranked next. Conversely, resources for gathering knowledge (e.g. lectures or textbooks) were of minor importance when studying for OSCEs. Reported studying time was lower for OSCEs compared to MCQ tests. The reported average grade, however, was better on OSCEs.

**Conclusions:**

The study findings suggest that the introduction of OSCEs shifted studying time. When preparing for OSCEs students focus on the acquisition of clinical skills and need less studying time to achieve the expected level of competence/performance, as compared to the MCQ tests.

**Electronic supplementary material:**

The online version of this article (10.1186/s12909-019-1570-6) contains supplementary material, which is available to authorized users.

## Background

Medical schools around the world have implemented objective structured clinical examinations (OSCEs) [1]. In an OSCE, students move through a series of stations where they have to perform specific clinical tasks within a time limit. The content domains to be assessed and the scoring scheme for the examination are defined in advance [2]. Since its first description in the mid-1970s [3], the OSCE has been the subject of countless papers [4]. A number of papers have shown that the OSCE is a valid and reliable assessment of a student’s clinical competence [5–8]. Papers have also shown that students accept the OSCE as a relevant and fair exam [9–11].

However, only a few studies exist on how the deployment of OSCEs affects students’ study behaviour. Newble and Jaeger [12], for instance, reported that work-based learning, textbooks, tutorials, and group activities were the predominant resources when studying for a clinical examination. Mavis [13] found that students focused on cognitive learning strategies, such as reviewing textbooks or class notes, when preparing for an OSCE. This study, however, was limited to the extent that the examined OSCE was a formative, and not a summative assessment, which may explain the different findings. Rudland and colleagues [14] identified that the OSCE fostered collaborative learning, but did not encourage students to spend more time learning in clinical settings. The disparities found in the literature suggest that the OSCE does not always drive student learning in the desirable way. The student study behaviour may rather depend upon of what is specifically assessed in the OSCEs, the purpose of the assessment (summative vs. formative), as well as other factors such as patient availability [15], advice given by the teachers or information from peers.

The aim of our study was to examine whether the introduction of OSCE assessments shifted the time students spend studying. We explored what resources were important for studying and how much time students spent when studying for OSCEs compared to traditionally used multiple choice question (MCQ) tests, and how they performed on the respective assessment format.

## Methods

### Context

We conducted the present study in the context of the amendment of the national medical licensure act carried out in 2002, which called for a more practice- and patient-oriented alignment of medical education in Germany [16]. Each of the 36 medical schools established before 2012 has a six-year curriculum. The curricula usually consist of two preclinical years followed by three clinical years and, finally, the clinical internship year. According to the guidelines of the medical licensure act, the clinical years comprise 41 predetermined courses entailing the full range of clinical areas or disciplines. During these courses, students have to pass summative (graded) in-house assessments designed by medical school members to be admitted to the national licensing examination. The medical licensure act sets the general framework of the undergraduate programme, but schools have considerable freedom to organise their own curricula. Thus, both the succession of the individual courses and their specific content, as well as the accompanying assessment strategy differ from one school to another.

Written assessments, in the form of MCQ tests, are still most commonly used during the clinical years in all German medical schools. The focus is on testing a student’s knowledge about diseases, involving pathogenesis, signs and symptoms, diagnostic approaches, and treatment strategies. In order to comply with the new legal requirements, medical schools have broadened their assessment repertoire to include performance related skills. By now, 33 of the 36 medical schools (92%) have introduced at least one summative OSCE into their in-house assessment system used for the clinical curriculum [17]. In the held OSCEs, the main focus is on the performance domains physical examination, history taking, practical procedures, and communication skills. Passing the OSCE(s) is also a prerequisite for students to be admitted to the national licensing examination.

### Student population

In the academic year 2014/15, there were around 88,000 medical students in Germany. Almost $2/3$ of them (53,352 [61%]) were female students [18]. We surveyed medical schools on both the number of students per year and the timing when OSCEs occurred in the curriculum. With these data, we calculated the proportion of students during the clinical years or the clinical internship year who had exposure to a summative OSCE at slightly more than 32,700.

### Data collection

Between February and April of 2015, we conducted this study using the free software package SoSci Survey (www.soscisurvey.de). Due to privacy terms, we did not get access to the students’ email addresses. We therefore could not administer our web-based questionnaire to a selected sample of the population in study; but instead, asked the medical schools in Germany to advertise the survey on their websites or through messaging systems. All medical students of years 3, 4, 5, and 6, who had undertaken at least one summative OSCE, were eligible to participate in the study. Participation was voluntary and anonymous, and the respondents did not receive any incentive for completing the questionnaire. The study was in accordance with the ethical standards of our institutional review board (Ethics Committee of Jena University Hospital at Friedrich Schiller University).

### Design of the questionnaire

We first reviewed literature and conducted interviews with students to identify items that we could use for our study. Based on this knowledge, we developed a draft questionnaire. As a next step, we repeatedly pilot-tested and revised the draft for ensuring that respondents completed the survey in the intended manner. The final version of the questionnaire comprised two identical sections, the first for the OSCE and the second for the MCQs.

Each section contained a list of 12 study resources (Table 1). Participants rated their preferences in preparation for the respective assessment method on a 5-point scale, anchoring 1 (*not important*), 2 (*slightly important*), 3 (*moderately important*), 4 (*important*), and 5 (*very important*). Participants then answered two open-ended questions. Firstly, we prompted them to estimate the average total time they spent preparing for a single summative in-house OSCE or MCQ test. To improve ease of completion, we requested them to indicate their time spent in working days of about 8 h. Secondly, we asked them to report their overall average grade achieved on each of the two assessment methods. In this paper, we present the reported grades on a 4.0 grading scale ranging from 0.0 (failing grade) and 1.0 (lowest passing grade) to 4.0 (best possible grade). Finally, the questionnaire collected demographic details, involving gender, age, academic year (semester), and medical school affiliation.

**Table 1:** Students’ preferences of study resources when preparing for OSCEs and MCQ tests

Study resource	OSCEs	MCQ tests
	Mean (*SD*)	Mean (*SD*)
Physical examination courses	4.38 (0.82)	2.09 (1.07)
Class notes/logs	3.88 (1.11)	4.25 (0.95)
Skills lab	3.87 (1.21)	1.83 (0.97)
Group learning	3.61 (1.26)	2.57 (1.29)
Medical clerkships	3.58 (1.16)	2.48 (1.14)
Clinical work placements	3.56 (1.12)	2.37 (1.13)
Peer tutorials	3.53 (1.19)	2.15 (1.11)
Textbooks	3.17 (1.07)	3.97 (0.98)
Multimedia materials	3.05 (1.11)	3.32 (1.19)
Casebooks	2.90 (1.14)	2.42 (1.14)
Lectures	2.67 (1.08)	4.07 (1.03)
PBL courses	2.48 (1.14)	2.12 (1.09)

*PBL* problem-based learning, *SD* standard deviation

The responses were made on a 5-point scale with anchors 1 (*not important*), 2 (*slightly important*), 3 (*moderately important*), 4 (*important*), and 5 (*very important*)

Number of respondents, *n* = 1131

The questionnaire also included an 11-item set on the benefit of the OSCE or MCQs (at the beginning of each section). The results are presented in a separate paper recently published in GMS Journal for Medical Education [19]. All the questionnaire items can be found in Additional file 1.

### Data analysis

After sampling, we verified that each respondent had exposure to a summative OSCE by squaring the indicated semester or the day when completing our questionnaire with the specific curriculum of the relevant medical school. For carrying out the statistical analysis, we used IBM SPSS Statistics for Windows, Version 24 (IBM Corp., Armonk, NY, USA). We performed descriptive statistics and used a paired *t*-test to compare participants’ responses on the open-ended questions between the OSCE and MCQs. We calculated Cohen’s *d* as a measure of effect size from the *t*-statistic (*t*-value, group size, and Pearson’s correlation coefficient). To determine whether the medical school had an influence on the results, we conducted a univariate ANOVA using the mean of the responses for the two assessment methods (excluding missing data) as the dependent variable and the medical school as the fixed factor. We considered *p* values below 0.05 statistically significant. When not stated otherwise, we present data as means with standard deviations in parentheses.

## Results

### Sample

The number of participants who completed the questionnaire was 1189. We removed 58 respondents, as either the demographic details were incomplete or we observed that those respondents had not yet been exposed to a summative in-house OSCE. Our analysis included 1131 respondents (777 female students [69%] vs. 354 male students [31%]) from 32 of the 33 medical schools (97%) that were holding summative OSCE(s). The sample represented all age groups of students, with a range from 19 to 45 years (median 25). Group sizes of years 4, 5, and 6 were similar (318 [28%], 338 [30%], and 303 [27%], respectively), while the proportion of year 3 due to less exposure to OSCEs was lower (172 [15%]). The number of respondents in each of the 33 medical schools varied between 0 and 123, with a mean of 34 responses per school, depending on how the individual schools advertised our study (websites vs. messaging systems).

### Study resources

All 1131 respondents included into the analysis completed the list on study resources. The ratings indicated that in preparation for OSCEs, students mostly preferred resources to acquire clinical skills. Physical examination courses (4.38 [0.82]) ranked first, followed by class notes/logs (3.88 [1.11]) and the skills lab (3.87 [1.21]). Medical clerkships (3.58 [1.16]) and clinical work placements (3.56 [1.12]), as well as group learning (3.61 [1.26]) and peer tutorials (3.53 [1.19]) ranked next. The ratings also showed that students attached moderate importance to resources for gathering knowledge, such as lectures (2.67 [1.08]) or textbooks (3.17 [1.07]), when studying for OSCEs.

Students’ preferences of resources for studying were different when they were preparing for MCQ tests. Resources to gather knowledge were most important, whereas those to acquire clinical skills were of minor importance. Class notes/logs (4.25 [0.95]), lectures (4.07 [1.03]), and textbooks (3.97 [0.98]) ranked first, second, and third, followed by multimedia materials (3.32 [1.19]). Table 1 shows the complete ratings on the list of study resources for both assessment methods.

### Studying time

We obtained valid responses from 1043 respondents. The reported number of hours spent for an OSCE was 66.5 (52.5) and 94.8 (71.5) for an MCQ test, respectively, which was significantly different (*t*[1042] = − 14.78, *p* <  0.01). Cohen’s *d* of 0.44 showed an effect size in the medium range.

The ANOVA revealed that the medical school had a significant influence on the duration of studying (*F*[31, 1011] = 5.40, *p* <  0.01, partial eta squared = 0.14). Table 2 includes the results on studying time by medical school affiliation. We found that respondents from about half of the medical schools (15/32 [47%]) reported a significantly lower time spent in preparation for an OSCE compared to an MCQ test, while the time spent did not differ significantly between the assessment methods for respondents of the other schools.

**Table 2:** Reported studying time for OSCEs and MCQ tests by medical school affiliation

Medical school	*n* ^b^	Time spent (h) for an OSCE^a^	Time spent (h) for an MCQ test^a^	*p* ^c^
		Mean	Mean	
VI	51	51.8	87.7	< 0.01
VII	38	41.6	76.5	< 0.01
VIII	38	50.6	109.5	< 0.01
X	108	89.1	146.2	< 0.01
XI	34	51.7	92.2	< 0.01
XII	32	54.3	120.6	< 0.01
XIV	72	59.9	82.0	< 0.01
XVI	40	52.5	105.8	< 0.01
XXXI	55	46.4	80.0	< 0.01
II	11	54.5	103.3	0.01
III	17	57.2	84.7	0.01
XXII	8	85.0	156.0	0.02
XXIII	18	44.1	95.6	0.02
XXIV	38	111.8	139.8	0.02
XXXII	50	47.9	63.7	0.04
IX	33	67.9	89.7	0.06
XXX	25	54.2	70.4	0.06
V	16	45.5	59.5	0.07
I	18	53.8	79.8	0.08
XXV	6	60.0	89.3	0.08
XXI	51	56.9	69.6	0.09
XXVIII	54	61.3	70.8	0.20
IV	57	78.3	89.2	0.22
XIII	15	70.9	92.8	0.32
XX	4	62.0	46.0	0.47
XXVI	31	77.2	85.5	0.47
XIX	6	121.3	149.3	0.48
XV	11	49.5	56.0	0.57
XXIX	5	67.2	77.6	0.60
XXVII	4	60.0	52.0	0.64
XVII	95	99.3	98.7	0.90
XVIII	2	64.0	60.0	0.91

^a^Average number of hours (h) students reported to study in preparation for a single summative in-house OSCE or MCQ test

^b^Number of responses

^c^A paired *t*-test was used

### Performance outcomes

From our respondents, 1111 replied to the question about performance outcomes. The reported average grade was 3.13 (0.62) on OSCEs and 2.84 (0.62) on MCQ tests, respectively. The difference was significant (*t*[1110] = 12.55, *p* <  0.01). Cohen’s *d* effect size was 0.47 indicating a medium effect.

There was a significant influence of the medical school on the OSCE and MCQ grades (*F*[31, 1079] = 6.48, *p* <  0.01, partial eta squared = 0.16). Table 3 shows the results on performance outcomes by medical school affiliation. Our analysis revealed that the reported grades were significantly better on OSCEs for respondents of almost half of the medical schools (14/32 [44%]), whereas the grades on MCQ tests were significantly better only in one school (XV).

**Table 3:** Performance outcomes in OSCEs and MCQ tests by medical school affiliation

Medical school	*n* ^b^	Average grade on OSCEs^a^	Average grade on MCQ tests^a^	*p* ^c^
		Mean	Mean	
I	22	3.52	2.90	< 0.01
V	16	3.38	2.47	< 0.01
VI	52	3.53	2.73	< 0.01
VII	42	3.61	3.00	< 0.01
IX	35	3.48	3.01	< 0.01
XI	34	3.26	2.86	< 0.01
XIV	72	3.12	2.76	< 0.01
XXI	54	2.89	2.60	< 0.01
XXIV	48	3.38	3.03	< 0.01
XXVIII	58	3.37	2.54	< 0.01
XXXI	57	3.38	2.81	< 0.01
XXVII	4	3.75	2.13	0.01
III	17	3.54	3.12	0.02
XIII	15	3.40	2.93	0.02
XXV	6	3.42	2.62	0.06
XXIX	5	3.60	3.00	0.07
XXXII	49	3.06	2.89	0.08
VIII	39	2.96	2.79	0.09
XX	4	2.50	3.00	0.09
XXII	8	3.25	3.00	0.10
XVI	43	3.24	3.08	0.13
XXX	23	2.98	2.72	0.16
XVII	105	3.05	2.95	0.19
XIX	8	3.19	2.84	0.21
X	122	2.60	2.66	0.38
XII	33	2.81	2.71	0.38
XVIII	2	3.25	2.75	0.50
XXIII	20	3.10	3.14	0.88
IV	62	2.96	2.97	0.92
XXVI	31	2.81	2.81	0.96
II	12	3.33	3.33	1.00
XV	13	2.85	3.48	0.01

^a^Overall average grade across all assessments of that kind as reported by the students. We used a 4.0 grading scale (minimum–maximum, 0.0–4.0)

^b^Number of responses

^c^A paired *t*-test was used

## Discussion

In response to the amendment of the national licensure act, German medical schools have incorporated OSCEs into their system of assessment. This nationwide study sought to address how the introduction of OSCEs has affected the time students spend studying. We identified that students use different strategies to prepare in advance of OSCE assessments than common MCQ tests. However, this finding was not surprising: When preparing for an assessment, students adapt their study behaviour (what and how they learn) to the assessment rather than to the learning objectives laid down in the curriculum. Both the content domains to be expected and the tasks required in the upcoming assessment influence student learning [20, 21], which has been described as pre-assessment learning effects of assessment [22]. Given the tasks being tested in the OSCEs (taking a history, examining a patient or carrying out a procedure), we therefore expected that students seek opportunities to rehearse the desired clinical skills. Although other authors have reported similar findings, they only examined one OSCE at a single institution and did not use a multi-centre approach [23, 24].

In conclusion, our findings depicted that the deployment of OSCEs has an impact on the students’ learning behaviour. In agreement with previous studies [23–25], the assessment tool encourages students to acquire clinical skills in, for example, physical examination, practical procedures or communication. The assessment also appears to motivate students – as compared to the MCQ tests – to focus more on studying in authentic learning environments and the community, both of which has been seen as important to support learning [26, 27].

If students prepare for an OSCE “designed to assess certain competencies or skills” [28]; vs. MCQs, which draw items from a much larger content domain, then they would probably need less study time to achieve the required learning outcomes. Our findings confirmed this assumption for the first time. We found that even though there were differences between schools, students spent less time preparing for an OSCE compared with an MCQ test, and yet performed well.

There has been evidence that scores achieved on the OSCE are strong predictors of a later clinical performance [29, 30]. However, good performance on the OSCE does not necessarily mean a student will have the same level of competence or performance in the clinical workplace. The simulated environment in which the OSCEs take place can influence the performance. Thus, a student might perform poorer when he/she is faced with unexpected, unusual circumstances in the real workplace [31]. It is important to keep this in mind when considering the (good) performance outcomes in OSCEs [28].

### Limitations

Our study has several limitations related to its sample. First, as we chose the sampling design of collecting data from every individual of the studied population by using advertisements, we could not retrace how many of the eligible students we approached. Therefore, we can report neither a response nor a non-response rate. Second, we found an overrepresentation of respondents from particular medical schools in our sample, which might have skewed the results. The varying degree to which the individual medical schools supported our study may have caused this fact. Nevertheless, the demographic profile of our sample reflected the general make-up of the medical student population in Germany and, in addition, we had a sufficiently large sample size for our analysis.

Another limitation of the study is that it relied on a self-reporting instrument to determine study resources, studying time, and performance outcomes leading to potential bias. This needs to be considered when interpreting the results.

## Conclusions

We conclude that the introduction of the OSCE assessment shifted the time students spend studying. In preparation for OSCEs, students focus their attention on acquiring the necessary clinical skills, and they need less study time to achieve the expected level of competence or performance compared with MCQ tests. This clearly confirms the value of adding the OSCE assessment to a testing programme, as it places the emphasis on the acquisition of practical skills in addition to knowledge.

## Additional file

Additional file 1: Questionnaire items (translated into English). (PDF 238 kb)

## Abbreviations

ANOVA: Analysis of variance
MCQs: Multiple choice questions
OSCEs: Objective structured clinical examinations
