# Supplementary material for: How the introduction of OSCEs has affected the time students spend studying: results of a nationwide study
Source: BMC Med Educ. 2019 May 15;19:146. doi: 10.1186/s12909-019-1570-6 (PMC6521539; doi:10.1186/s12909-019-1570-6)
Supplement: Supplementary file 1 — Questionnaire items (translated into English). (PDF 238 kb) [file 12909_2019_1570_MOESM1_ESM.pdf]

## Objective Structured Clinical Examination (OSCE)

**What does the OSCE do for you?**

| | *strongly disagree* | *disagree* | *neither agree nor disagree* | *agree* | *strongly agree* |
|---|---|---|---|---|---|
| OSCE gives me an understanding of medical care | | | | | |
| demonstrates the practices and principles of medical treatment | | | | | |
| gives me feedback on my performance level | | | | | |
| reveals my strengths in medical practice | | | | | |
| reveals my weaknesses in medical practice | | | | | |
| shows me gaps in my education | | | | | |
| enhances my problem-solving and decision-making abilities | | | | | |
| promotes my theoretical knowledge | | | | | |
| reflects the requirements of the medical profession | | | | | |
| allows me to assess my own ability to work as a medical professional | | | | | |
| helps me with my speciality choice | | | | | |

## Objective Structured Clinical Examination (OSCE)

**How important are each of the following resources to prepare for OSCEs?**

| | *not important* | *slightly important* | *moderately important* | *important* | *very important* |
|---|---|---|---|---|---|
| Physical examination courses | | | | | |
| Skills lab | | | | | |
| Clinical work placements | | | | | |
| Medical clerkships | | | | | |
| Peer tutorials | | | | | |
| Group learning | | | | | |
| PBL courses | | | | | |
| Multimedia materials | | | | | |
| Lectures | | | | | |
| Textbooks | | | | | |
| Casebooks | | | | | |
| Class notes/logs | | | | | |

## Objective Structured Clinical Examination (OSCE)

**How much time do you spend preparing for an OSCE?**

Average total preparation time for **one** summative OSCE, including course attendance

*(one day = approx. 8 working hours)*

days

## Objective Structured Clinical Examination (OSCE)

**What are your OSCE results?**

Your average OSCE result

[grade]

## Multiple Choice Questions (MCQs)

**What does the MCQ format do for you?**

| | *strongly disagree* | *disagree* | *neither agree nor disagree* | *agree* | *strongly agree* |
|---|---|---|---|---|---|
| MCQ format gives me an understanding of medical care | ○ | ○ | ○ | ○ | ○ |
| demonstrates the practices and principles of medical treatment | ○ | ○ | ○ | ○ | ○ |
| gives me feedback on my performance level | ○ | ○ | ○ | ○ | ○ |
| reveals my strengths in medical practice | ○ | ○ | ○ | ○ | ○ |
| reveals my weaknesses in medical practice | ○ | ○ | ○ | ○ | ○ |
| shows me gaps in my education | ○ | ○ | ○ | ○ | ○ |
| enhances my problem-solving and decision-making abilities | ○ | ○ | ○ | ○ | ○ |
| promotes my theoretical knowledge | ○ | ○ | ○ | ○ | ○ |
| reflects the requirements of the medical profession | ○ | ○ | ○ | ○ | ○ |
| allows me to assess my own ability to work as a medical professional | ○ | ○ | ○ | ○ | ○ |
| helps me with my speciality choice | ○ | ○ | ○ | ○ | ○ |

## Multiple Choice Questions (MCQs)

**How important are each of the following resources to prepare for MCQ tests?**

| | *not important* | *slightly important* | *moderately important* | *important* | *very important* |
|---|---|---|---|---|---|
| Physical examination courses | ○ | ○ | ○ | ○ | ○ |
| Skills lab | ○ | ○ | ○ | ○ | ○ |
| Clinical work placements | ○ | ○ | ○ | ○ | ○ |
| Medical clerkships | ○ | ○ | ○ | ○ | ○ |
| Peer tutorials | ○ | ○ | ○ | ○ | ○ |
| Group learning | ○ | ○ | ○ | ○ | ○ |
| PBL courses | ○ | ○ | ○ | ○ | ○ |
| Multimedia materials | ○ | ○ | ○ | ○ | ○ |
| Lectures | ○ | ○ | ○ | ○ | ○ |
| Textbooks | ○ | ○ | ○ | ○ | ○ |
| Casebooks | ○ | ○ | ○ | ○ | ○ |
| Class notes/logs | ○ | ○ | ○ | ○ | ○ |

## Multiple Choice Questions (MCQs)

**How much time do you spend preparing for an MCQ test?**

Average total preparation time for **one** summative MCQ test, including course attendance

*(one day = approx. 8 working hours)*

[ ] days

## Multiple Choice Questions (MCQs)

**What are your MCQ test results?**

Your average MCQ test result [ ] [grade]

## Demographics

| | Female | Male |
|---|---|---|
| What is your gender? | ○ | ○ |

How old are you? [ ] years

Which semester are you in? [Please select] ▼

What medical school are you from? [Please select] ▼
